# Interplay between noise-induced sensorineural hearing loss and hypertension: pathophysiological mechanisms and therapeutic prospects

**DOI:** 10.3389/fncel.2025.1523149

**Published:** 2025-04-07

**Authors:** Carola Y. Förster, Sergey Shityakov, Stavros Stavrakis, Verena Scheper, Thomas Lenarz

**Affiliations:** ^1^Department of Anesthesiology, Intensive Care, Emergency and Pain Medicine, University Hospital Würzburg, Würzburg, Germany; ^2^Laboratory of Chemoinformatics, Infochemistry Scientific Center, ITMO University, Saint Petersburg, Russia; ^3^Cardiovascular Section, Department of Medicine, University of Oklahoma Health Sciences Center, Oklahoma, OK, United States; ^4^Department of Otolaryngology, Hannover Medical School, Hannover, Germany; ^5^Cluster of Excellence “Hearing4all”, German Research Foundation, Hannover, Germany

**Keywords:** noise-induced hearing loss, sensorineural hearing loss, blood-labyrinth barrier, hypertension, vagus nerve stimulation, taVNS, network pharmacology

## Abstract

More than 5% of the global population suffers from disabling hearing loss, primarily sensorineural hearing loss (SNHL). SNHL is often caused by factors such as vascular disorders, viral infections, ototoxic drugs, systemic inflammation, age-related labyrinthine membrane degeneration, and noise-induced hearing loss (NIHL). NIHL, in particular, leads to changes in blood-labyrinth-barrier (BLB) physiology, increased permeability, and various health issues, including cardiovascular disease, hypertension, diabetes, neurological disorders, and adverse reproductive outcomes. Recent advances in neuromodulation and vector-based approaches offer hope for overcoming biological barriers such as the BLB in the development of innovative treatments. Computational methods, including molecular docking, molecular dynamics simulations, QSAR/QSPR analysis with machine/deep learning algorithms, and network pharmacology, hold potential for identifying drug candidates and optimizing their interactions with BLB transporters, such as the glutamate transporter. This paper provides an overview of NIHL, focusing on its pathophysiology; its impact on membrane transporters, ion channels, and BLB structures; and associated symptoms, comorbidities, and emerging therapeutic approaches. Recent advancements in neuromodulation and vector-based strategies show great promise in overcoming biological barriers such as BLB, facilitating the development of innovative treatment options. The primary aim of this review is to examine NIHL in detail and explore its underlying mechanisms, physiological effects, and cutting-edge therapeutic strategies for its effective management and prevention.

## 1 Introduction

### 1.1 Noise-induced hearing loss

NIHL is a very prominent type of SNHL that is particularly pronounced among adults. Two different conditions must be separated: acute acoustic trauma caused by, e.g., blast exposure and hearing loss as a consequence of chronic noise exposure.

In this review, we focus on NIHL resulting from ongoing noise trauma. It is the result of ongoing high levels of occupational, military, and recreational noise, which are the most common triggers (Wang et al., 2020; Zhou and Zhang, 2024).

Specifically, young people are at risk of developing NIHL because of the increasing use of headphones to enjoy music (Imam and Hannan, 2017; Jiao et al., 2022).

Individuals exposed to sound levels exceeding 85 dB for more than 5 h per week are at risk of experiencing permanent hearing damage over time. Notably, the relationship between occupational noise-induced hearing loss and the associated risk of various adverse health outcomes varies with frequency, among other factors (Imam and Hannan, 2017; Jiao et al., 2022).

Taken together, the World Health Organization reports that loud sound exposure potentially causes NIHL in more than 600 million people worldwide (Chadha et al., 2021; Alberti et al., 1979).

This paper provides an overview of NIHL, focusing on its pathophysiology; its impact on membrane transporters, ion channels, and BLB structures; and associated symptoms, comorbidities, and emerging therapeutic approaches. Recent advancements in neuromodulation and vector-based strategies show great promise in overcoming biological barriers such as BLB, facilitating the development of innovative treatment options. The primary aim of this review is to examine NIHL in detail and explore its underlying mechanisms, physiological effects, and cutting-edge therapeutic strategies for its effective management and prevention.

## 2 Auditory structures and functions impacted by noise

Sounds at or below 70 A-weighted decibels (dBA), even after long exposure, are unlikely to cause hearing loss. However, long or repeated exposure to sounds at or above 85 dBA can cause hearing loss. The louder the sound is, the shorter the amount of time it takes for NIHL to occur. Ongoing acoustic exposure to intense sound can induce a level of damage that is accompanied by a temporary threshold shift (TTS), acute changes in hearing sensitivity that recover over time, or impairments accompanied by a permanent threshold shift (PTS), a loss that does not recover to preexposure levels.

The severity of NIHL is influenced by various environmental factors, including the characteristics of noise and the duration of exposure. The characteristics of noise, such as frequency, intensity, and temporal pattern, play crucial roles in the extent of auditory damage. Compared with continuous, steady-state noises such as those from industrial machinery, impulsive noises, such as explosions or gunfires, are known to cause more immediate and severe cochlear damage (Le Prell et al., 2007). Impulsive noise produces a rapid, high-intensity sound pressure level, leading to mechanical disruption of cochlear structures and immediate hearing threshold shifts (Kujawa and Liberman, 2009; Gratias et al., 2021). Moreover, the frequency of noise is a critical determinant of the location and severity of cochlear damage. High-frequency sounds (above 4 kHz) are particularly damaging to the basal turn of the cochlea, which is responsible for high-frequency sound processing (Nordmann et al., 2000). This damage can lead to high-frequency hearing loss, which often precedes damage in lower frequency regions, thus affecting speech comprehension and communication abilities (Liberman et al., 2015). In addition to the noise characteristics, the duration of the exposure is relevant. Prolonged exposure to noise is directly correlated with the severity of NIHL. The relationship between exposure duration and hearing loss is cumulative; even moderate levels of noise can lead to significant auditory damage if exposure persists over time. The World Health Organization (WHO) identified 85 dB as the threshold above which prolonged exposure can lead to permanent hearing damage (Schubert et al., 2023).

The interplay between noise type and exposure duration further complicates the pathophysiology of NIHL. Continuous noise exposure at lower intensities may cause less immediate damage but can result in significant cumulative effects over time. Conversely, short-duration exposure to impulsive, high-intensity noise can lead to acute cochlear trauma including synaptopathy and immediate hearing loss (Seidman and Standring, 2010; Gratias et al., 2021).

Chronic noise exposure leads to both TTS and PTS. TTS, characterized by reversible hearing loss after noise exposure, may become PTS with continued exposure, resulting in irreversible damage to cochlear hair cells (Henderson et al., 2006). Kujawa and Liberman (2009) demonstrated that repeated episodes of TTS could cause progressive synaptic degeneration even if the threshold shifts initially recover, indicating that subclinical damage accumulates over time and contributes to long-term hearing loss. However, despite this previous assumption, recurrent episodes of TTS may not be related to PTS in the long term because the two conditions are characterized by different pathogenetic mechanisms.

### 2.1 Mechanisms of TTS

TTS is defined as at least 10 dB of threshold elevation at one or more frequencies between 2 and 4 kHz. This threshold shift may reach 50 dB (Liberman, 2016). TTS lasts from minutes to days, depending on the pathophysiology of the damage. Low-level TTS is mediated by ion channels that are activated by extracellular ATP (Housley et al., 2013). The relevant ATP receptor P2RX2 is a nonselective cation channel expressed in cochlear hair cells (HCs) and epithelial cells lining the scala media. Noise stimulates local ATP release in the cochlea, and ATP opens the channels, which then shunts the endocochlear current away from the HC transduction channel (Morton-Jones et al., 2015; Thorne et al., 2004). Higher levels of TTS (up to 50 dB) are due to additional mechanisms, such as uncoupling of the outer HC stereocilia from the tectorial membrane (Nordmann et al., 2000) and swelling of the afferent endings underneath the inner HCs, suggestive of excitotoxicity due to the release of excessive glutamate from overstimulated HCs (Puel et al., 1998). Other evidence suggests that damaging levels of noise lead to metabolic overstimulation and subsequent generation of free radical species (Henderson et al., 2006; Shi and Nuttall, 2003) like reactive oxygen species (ROS) and reactive nitrogen species (RNS).

### 2.2 Mechanisms of PTS

When exposed to sufficiently loud and long-lasting noise, the ability of the cochlea to recover becomes overwhelmed, leading to irreversible hearing loss. This type of hearing loss is mainly linked to damage to and loss of cochlear HCs, but damage to neurons and other parts of the cochlea can also contribute to PTS (Kurabi et al., 2017). This damage can directly disrupt stereocilia (Slepecky, 1986), which diminishes or completely stops their function. In severe cases, damage can even affect the overall structure of the sensory part of the cochlea, disrupting HCs and their support cells. This type of damage can also create a connection between two fluid-filled areas in the cochlea, endolymph, and perilymph, allowing excessive levels of potassium to reach the basal poles of remaining intact HCs, leading to their death. However, damaging levels of noise begin well below the threshold of such frank mechanical damage. The majority of NIHL is caused by damage to HCs through biochemical processes that occur within the cells themselves via ROS production, as in the case of TTS.

In the cochlea, noise exposure generates excessive ROS and RNS, leading to oxidative stress, lipid peroxidation, and DNA damage, which trigger apoptosis in hair cells and disrupt the BLB (Yamashita et al., 2005; Le Prell et al., 2007; Mao and Chen, 2021; Natarajan et al., 2024). These molecules also induce inflammation, exacerbating cochlear damage (Mao and Chen, 2021; Natarajan et al., 2024). At a systemic level, ROS and RNS contribute to endothelial dysfunction, sympathetic nervous system activation, and renin–angiotensin–aldosterone system (RAAS) activation, all of which increase vascular resistance and promote hypertension (Yamashita et al., 2005). The interaction between oxidative stress in the cochlea and systemic vascular dysfunction suggests a potential mechanistic link between NIHL and hypertension. This connection provides insights for therapeutic interventions aimed at targeting oxidative pathways (Yamashita et al., 2005; Le Prell et al., 2007).

### 2.3 TTS, PTS, and free radical impact

The cascade of events initiated by ROS induction entails lipid peroxidation within the cochlea, thereby giving rise to the generation of highly noxious metabolic byproducts. While the products of lipid peroxidation alone can induce apoptosis, lipid peroxidation byproducts possessing vasoactive properties, such as isoprostanes, may contribute to a diminished blood supply to the cochlea (Ohinata et al., 2000; Thorne et al., 1987; Seidman et al., 1999). Noise-induced ischemia and subsequent reperfusion might further potentiate the generation of ROS in a positive feedback loop (Kurabi et al., 2017). Furthermore, ROS-mediated mechanisms involve the induction of inflammatory responses, including the production of proinflammatory cytokines such as interleukin-6 (IL-6) and tumor necrosis factor *α* (TNFα), which themselves have the ability to damage the cochlear structure and function (Tan et al., 2016).

## 3 The role of the stria vascularis in NIHL

Recent investigations highlight the role of the stria vascularis (SV), located in the lateral wall of the cochlea, in the pathogenesis of NIHL (Yu et al., 2021). A previous study revealed that loud noise exposure could lead to a reduced vessel diameter but, concomitantly, an increase in vascular permeability in the SV (Quill et al., 2015) and increased macromolecular transport, which could decrease the endocochlear potential (EP) (Suzuki et al., 2002). However, the underlying mechanism remains unclear and might be related to the formation of excess ROS, RNS, the release of proinflammatory cytokines, and ultimately excitotoxicity, as detailed above (com. 1.1.1. – 1.1.3.).

**Figure 1:**
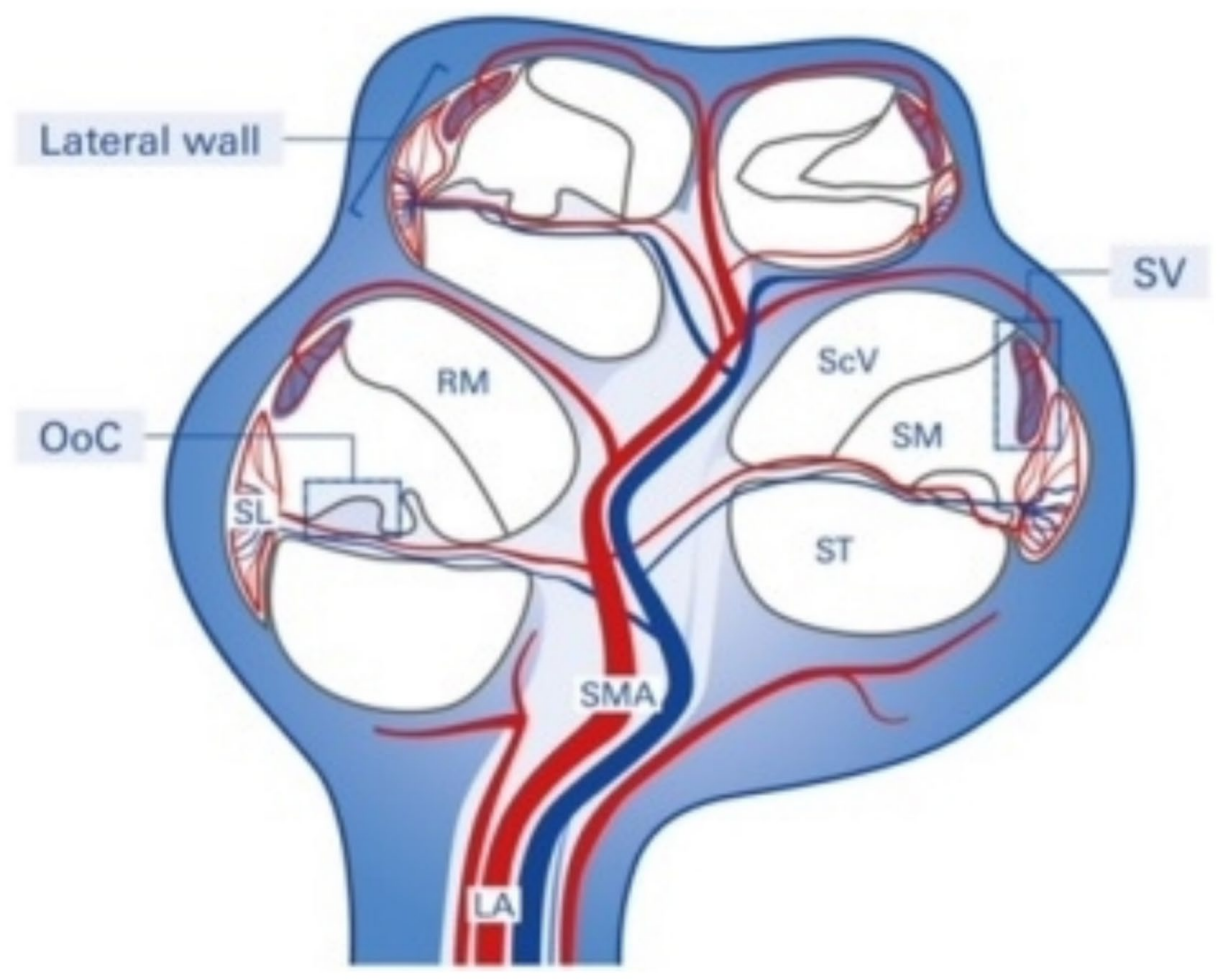
Structure and blood supply of the cochlea. The spiral modiolar artery supplies the OoC of the modiolus and forms the capillaries of the spiral ligament and stria vascularis in the cochlear lateral wall. SMA, spiral modular artery; SL, spiral ligament; SV, stria vascularis; OoC, organ of Corti; ScV, scala vestibuli; SM, scala media; ST, scala tympani; RM, Reissner membrane; LA, labyrinthinth artery.

**Figure 2:**
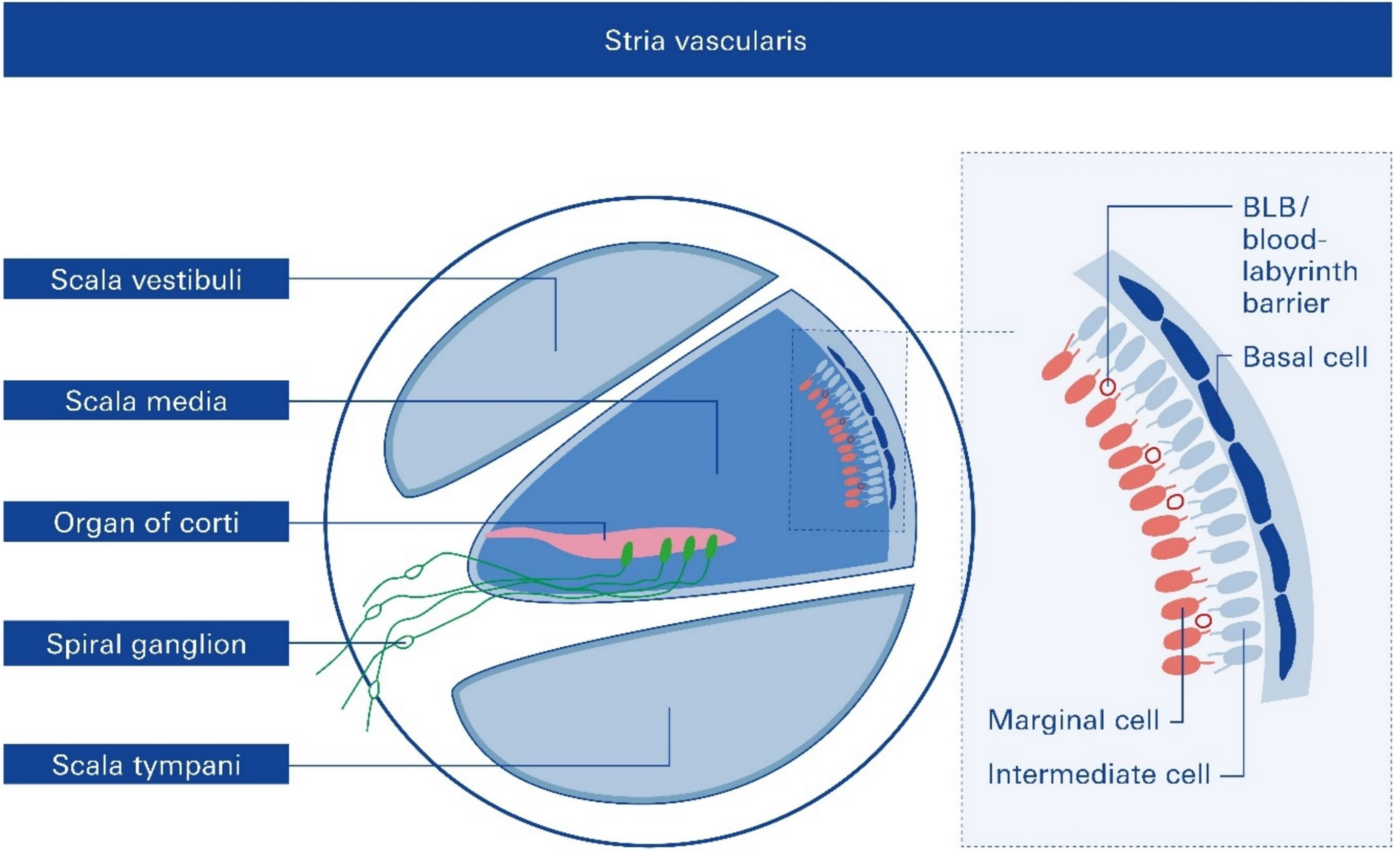
Cross section of one cochlear turn and rough structure of the stria vascularis, SV. Structural and functional damage to the SV mediated by noise trauma, specifically to the endothelial blood–labyrinth barrier (BLB), causes hearing loss, but the underlying mechanisms remain mostly unclear.

The SV is derived from the spiral modiolar artery supplying the organ of Corti (OoC) and primary auditory neurons (Figure 1) and is mainly composed of marginal, intermediate, and basal cells and endothelial cells forming the so-called strial BLB (Figure 2).

Anatomically, the blood supply to the cochlea comes from the common cochlear artery, which divides into the spiral modiolar artery and the vestibulocochlear artery. The spiral modiolar artery supplies the apical turns of the cochlea, and the cochlear branch of the vestibulocochlear artery supplies the basal turns of the cochlea (Figure 1). The spiral modiolar artery supplies the OoC and primary auditory neurons of the modiolus and forms the capillaries of the spiral ligament and *stria vascularis* in the cochlear lateral wall. Strial capillaries are nonfenestrated with tight junctions between adjacent endothelial cells and a decreasing rate of entry into perilymph from blood by compounds of increasing molecular weight (Jahnke, 1980; Juhn et al., 1981), forming a barrier that separates intrastrial fluids from blood, the BLB. Historically, the concept of the strial BLB in the inner ear originated from the observed difference in the chemical composition of blood and inner ear fluids (Figure 2).

The significant functions of the SV are to (i) generate the endocochlear potential (EP), which is essential for audition, (ii) secrete endolymph, and (iii) maintain cochlear homeostasis by controlling ion homeostasis and substance exchange between the blood and the interstitial space in the cochlea at the level of the endothelial BLB (Figure 2).

### 3.1 The strial blood–labyrinth barrier provides a functional contribution to SNHL pathologies

The strial BLB is a highly specialized network of capillaries that tightly controls the permeability of the capillaries and controls macromolecular exchange between the blood and the interstitial space in the cochlea (Forster et al., 2022; Forster et al., 2005). The strial BLB thus maintains cochlear homeostasis and protects the cochlea from blood-borne potentially ototoxic endobiotics and xenobiotics.

The strial BLB is fundamentally similar to the blood–brain barrier (BBB), which separates brain interstitial fluid from blood (Figure 1). The BLB separates the inner ear fluid compartments (perilymph and endolymph) from capillaries of the vasculature and is composed of vascular endothelial cells coupled together by tight junctions. BLB is critical for maintaining ionic homeostasis in inner ear fluid and preventing the entry of deleterious substances into the inner ear. Recent studies have implicated the loss of integrity of the BLB in several inner ear pathologies, including acoustic trauma, infection, ototoxin-induced hearing loss, and age-related hearing loss (presbycusis).

**Figure 3:**
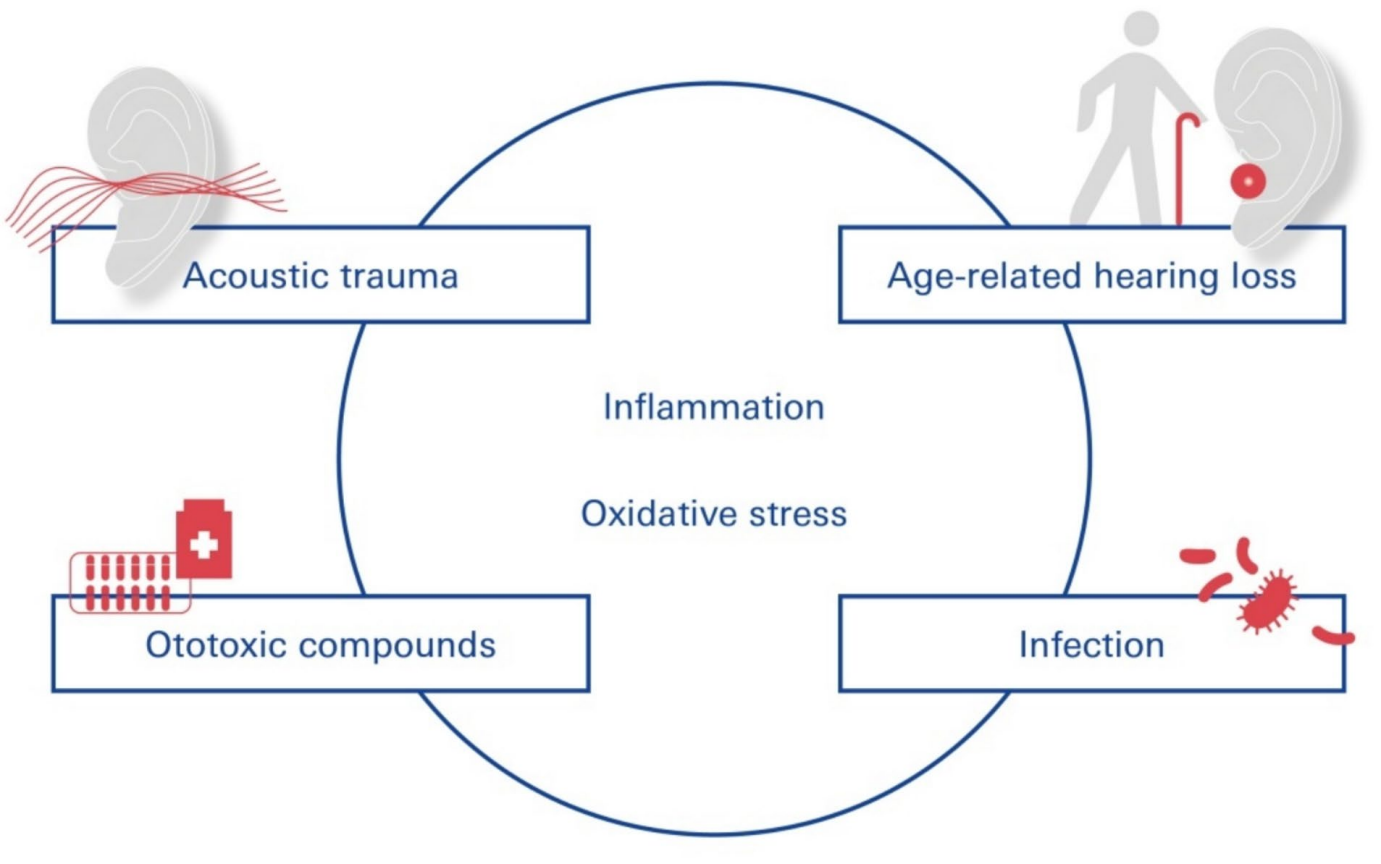
SNHL—functional contribution of the blood-labyrinth-barrier. Pathogens leading to SNHL include vascular disorders, viral infections, noise trauma, ototoxic drug exposure, and age-related degeneration of the labyrinthine membrane.

In this context, loud sounds affect almost all cochlear cell types and induce inflammation and drug-induced cochleotoxicity (Li et al., 2011; Vethanayagam et al., 2016). Acoustic trauma (Suzuki et al., 2002) involves several external and intrinsic factors that can profoundly modulate the permeability of the BLB (Taylor et al., 2008), increasing its permeability and subsequent uptake of drugs indirectly by inducing inflammation and degeneration of sensory cells and auditory neurons (Figure 3).

Different disease states comorbid with NIHL can alter BLB physiology and increase its permeability through inflammation and oxidative stress: cochlear inflammation (Kastenbauer et al., 2001; Hirose et al., 2014) caused by acoustic trauma, in turn, contributes to the degeneration of cochlear sensory cells.

## 4 Spectrum of NIHL-associated disorders

Repeated overexposure to noise at or above 85 dB can cause permanent hearing loss, tinnitus, and difficulty in understanding speech in noise. It is also associated with cardiovascular disease, depression, cognitive dysfunction, balance problems, and lower income (Themann and Masterson, 2019). Patients with untreated NIHL are susceptible to fatigue, difficulty communicating, social isolation, and stress as a result of their illness (Reiss and Price, 1996).

First, and very obviously, hearing impairment per se hinders effective communication, leading to misunderstanding and social withdrawal. This may lead to feelings of loneliness and depression. The frustration associated with impaired communication can exacerbate depressive symptoms. Studies have shown that untreated hearing loss increases the likelihood of depression, highlighting the importance of early intervention and rehabilitation of hearing loss (Mener et al., 2013; Lawrence et al., 2020). Additionally, noise affects cognition, as demonstrated by Thompson et al. (2022), who reported high-quality evidence for an association between environmental noise and cognitive impairment in middle-to-older adults and moderate-quality evidence for an association between aircraft noise and reading and language in children. Another aspect affecting mental health is that individuals with hearing loss often exert additional effort to comprehend speech, resulting in increased cognitive load and listening fatigue. This heightened mental exertion can lead to decreased concentration and overall exhaustion, affecting daily functioning and quality of life (Shukla et al., 2020). The abovementioned communication challenges and mental load may lead to reduced productivity and employment opportunities. This economic impact is particularly pronounced in occupations requiring effective verbal interactions, where hearing impairment may limit career advancement and earning potential.

The inner ear houses both the auditory and vestibular systems; thus, damage from excessive noise exposure can affect balance. Research has revealed a correlation between NIHL and vestibular dysfunction, which manifests as vertigo, postural instability, and motion intolerance (Natarajan et al., 2024).

**Figure 4:**
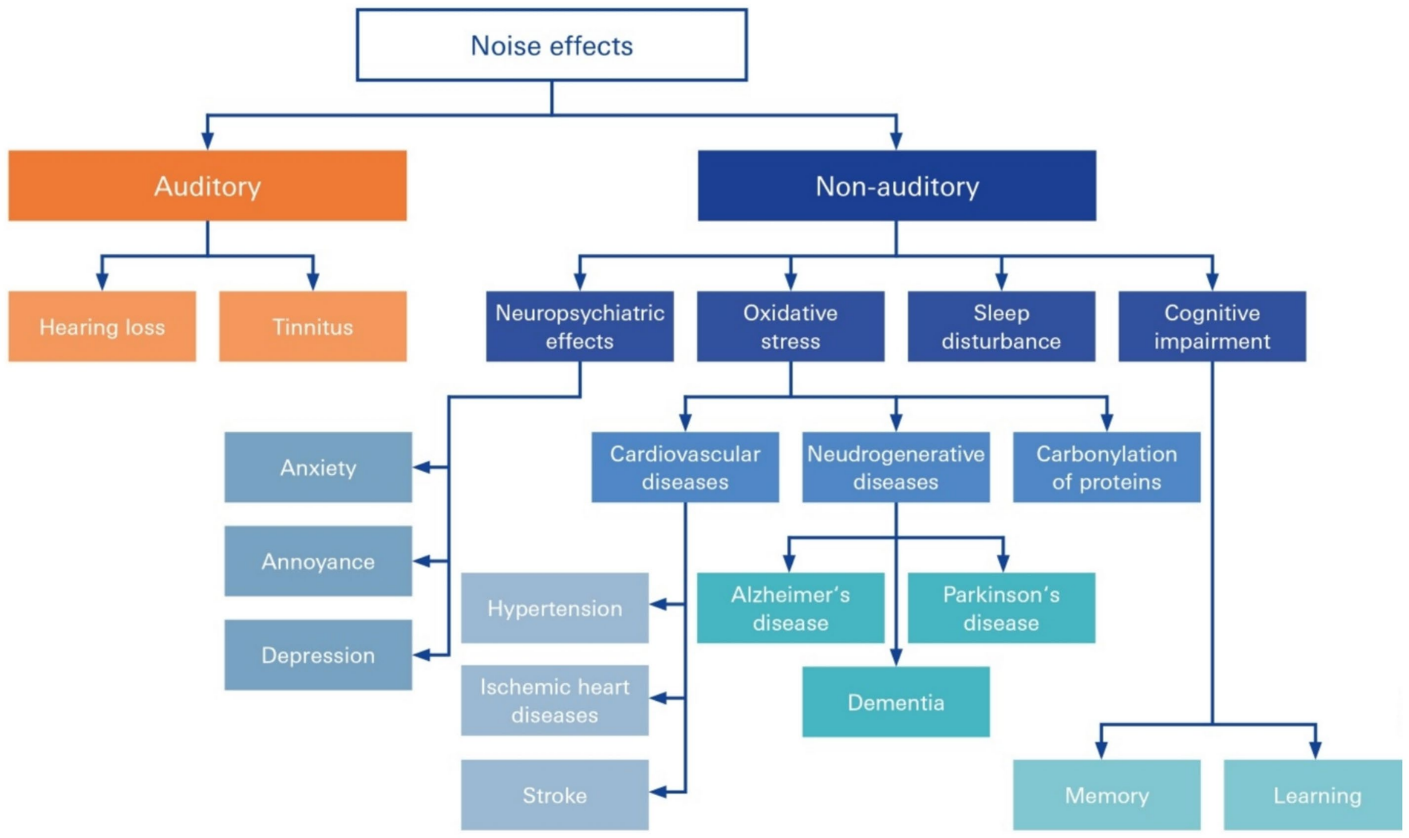
Auditory and nonauditory effects of noise overexposure. Noise occurs in everyday life and can lead to both auditory and nonauditory adverse health effects, including hearing loss, tinnitus, neuropsychiatric effects, and cognitive impairment, and can be secondary to triggered circuit conditions, such as cardiovascular and neurodegenerative disease and hypertension.

Damage to the SV in NIHL has been recognized to be at the root of many common disorders and syndromic diseases accompanied by NIHL, many of which are cerebro- and cardiovascular diseases. The accumulation of noise-induced damage to the inner ear is a key trigger of age-related hearing loss and cognitive decline (Förster et al., 2019; Förster et al., 2022; Nagai et al., 2021; Shityakov et al., 2021a; Förster et al., 2020; Nagai et al., 2021), tinnitus and even diminished learning and cognitive abilities in children and adolescents (Manukyan, 2022) (Figure 4). Chronic, subjective tinnitus, an auditory phantom sensation in the absence of physical stimuli, can be triggered by a variety of factors that may act synergistically (Knipper et al., 2020).

The major cause of cochlear damage resulting in deafferentiation is environmental noise overexposure (Chen et al., 2021; Hickman et al., 2021).

Notably, in addition to noise-induced tinnitus (Hayes et al., 2023; Johns et al., 2023), approximately 64% of patients with NIHL exhibit comorbidities, and the most common condition is hypertension.

## 5 Noise above the permissive threshold may beget autonomic changes and hypertension

**Figure 5:**
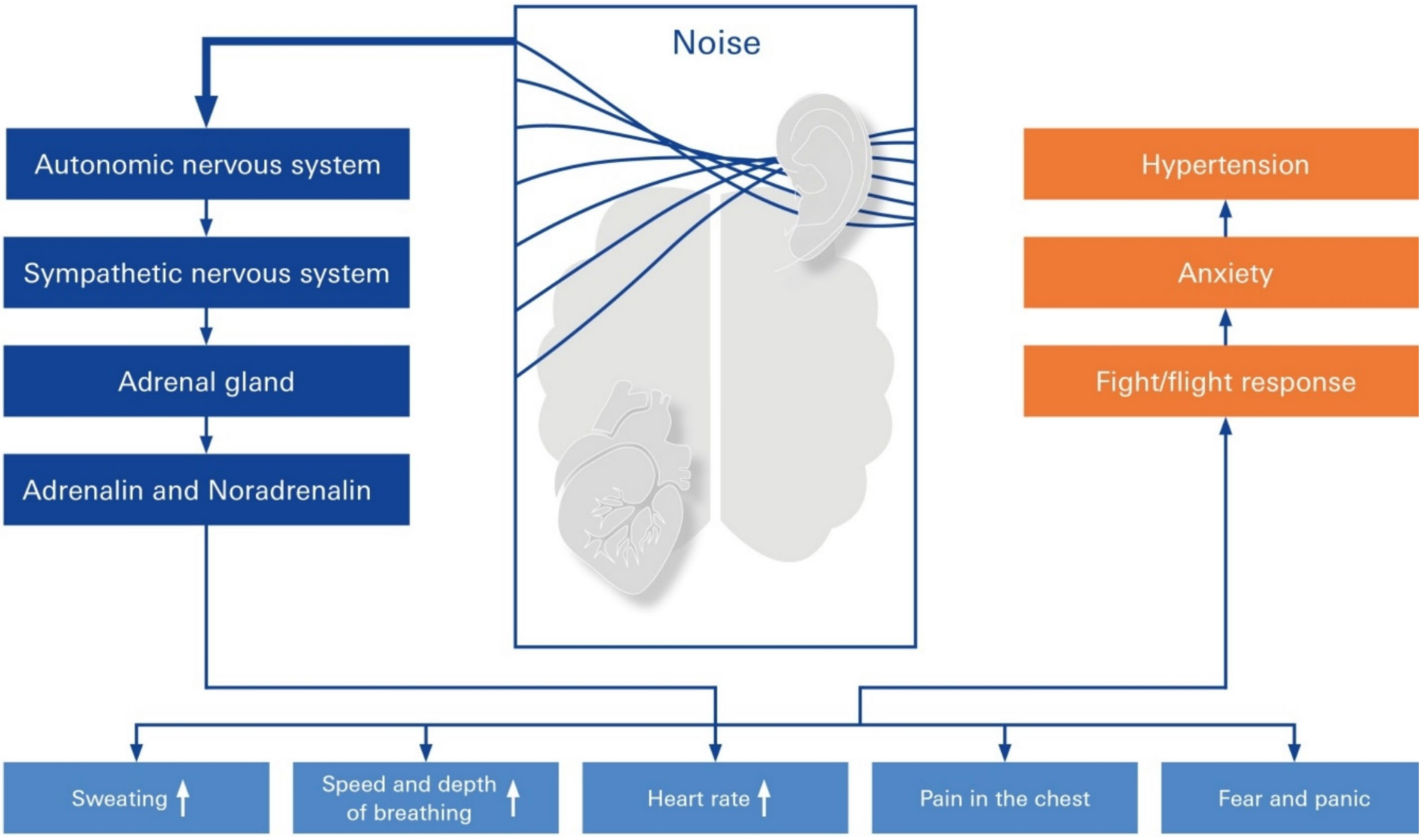
Stress response elicited by elevated noise. Pathways of elevated noise action lead to hypertension through mental stress, autonomic nervous system changes in sympathetic imbalance, anxiety, and neurohormonal mechanisms. The elevated risk factor is noise exposure because of a primary rudimentary stress reaction, which is mediated either by activation of the sympathetic nervous system or the hypothalamic–pituitary–adrenal (HPA) axis, resulting in fight/flight response, anxiety, and hypertension.

There was a greater incidence of hypertension among patients with sudden sensorineural hearing loss (SSNHL) than among 54,946 matched controls (Saba et al., 2023). Sustained noise exposure may result in health effects related to stress after acute noise exposure (Liu et al., 2022) (Figure 5). Specifically, there appears to be an increased risk of hypertension associated with noise-induced high-frequency hearing loss, and the risk varies by age and work experience (Liu et al., 2022): the presence of elevated noise exposure was associated with an increased risk of hypertension in a meta-analysis of 32 studies involving 264,678 participants. A significant dose–response relationship between noise exposure and hypertension was found (Fu et al., 2017).

Several epidemiological studies have revealed strong associations between occupational noise exposure, noise-induced hearing loss and hypertension (Zhang et al., 2022). The HYENA Study (Hypertension and Exposure to Noise Near Airports) investigated the impact of aircraft noise on the blood pressure of residents living near six major airports. The results revealed that an increase in nocturnal aircraft noise of 10 dB increased the risk of hypertension by 14% (Jarup et al., 2008). One European long-term study observed over 41,000 individuals in five countries over a period of up to nine years. The results indicated that both air pollution and road traffic noise were significantly associated with the development of hypertension. The risk existed even at levels below the current EU limits. Chang and colleagues examined the impact of occupational noise exposure, including blood pressure, on vascular health over a 24 h period. The results indicated that workers exposed to high levels of noise presented altered vascular properties, such as increased blood pressure, suggesting a link between noise exposure, hearing loss, and hypertension (Chang et al., 2007). The same team investigated the associations between high-frequency hearing loss, which is indicative of chronic noise exposure, and hypertension among male workers. The findings suggested a significant correlation between occupational noise exposure leading to hearing loss and elevated blood pressure levels (Chang et al., 2007).

Although causation cannot be inferred from such studies, they provide substantial evidence that environmental noise should be avoided if possible. Current evidence suggests that exposure to noise levels above 85 dB leads to autonomic changes and alterations in blood pressure (Wojciechowska et al., 2022; Thiesse et al., 2020; Gangwar et al., 2023). In addition to autonomic changes, environmental noise above 85 dB induced endothelial dysfunction, as did increases in plasma noradrenaline and angiotensin II levels in mice (Munzel et al., 2017). Furthermore, environmental noise exposure leads to increases in stress hormone levels, thereby triggering inflammatory and oxidative stress pathways and inducing endothelial dysfunction, all of which could lead to hypertension (Munzel et al., 2018). Future studies should explore whether targeting these pathways prevents the development of hypertension in those exposed to occupational and/or environmental noise.

## 6 Novel therapies to treat NIHL

It has been consistently demonstrated that noise, be it environmental, leisure-derived, or occupational, above the permissible limit of 85 dB leads to autonomic changes and alterations in blood pressure (Wojciechowska et al., 2022; Thiesse et al., 2020; Gangwar et al., 2023). These data were corroborated by animal data showing that environmental noise above 85 dB in a mouse model induced endothelial dysfunction, as well as increases in plasma noradrenaline and angiotensin II levels (Munzel et al., 2017). In the following section, we provide incentives for the future clinical translation of NIHL for prevention and therapy.

### 6.1 Computer-assisted drug design and new modeling paradigms for NIHL

Computational modeling and simulation of drug permeation through biological barriers is a promising approach for developing novel drug therapies for pathological processes such as NIHL. By providing insights into drug behavior, barrier characteristics, and physiological conditions, in silico modeling can aid in the design of more effective and targeted drug delivery strategies across the BLB.

Recent advancements in computational modeling and simulation techniques have enabled researchers to better understand the underlying mechanisms of drug transport and identify potential therapeutic targets and drug transporters at barrier interfaces, such as BBB-ChT (blood–brain barrier choline transporter) and P-gp (P-glycoprotein) (Shityakov and Forster, 2013; Shityakov and Forster, 2014a,b). However, most simulation models have been developed for the BBB, which separates the brain and blood compartments.

On the other hand, the complex nature of the auditory system and the BLB has posed significant challenges for traditional drug discovery methods, as there are limited experimental data available to develop novel approaches such as structure–activity–property relationship (QSAR and QSPR) models. The BLB is a specialized structure that separates the inner ear from the systemic circulation and is composed of tightly packed endothelial cells that restrict the diffusion of molecules and ions across the barrier. This presents a significant challenge for drug delivery through the blood into the perilymph of the inner ear, where traditional systemic drug administration is ineffective (Le and Blakley, 2017). Owing to this, permeation partitioning coefficients, such as the logBB (blood–brain partitioning coefficient) and logPS (permeability–surface area product), are still needed for the BLB (logBL–blood–labyrinth partitioning coefficient) to develop accurate regression models already implemented for the BBB (Shityakov and Forster, 2013; Shityakov et al., 2013; Shityakov et al., 2021b; Shityakov et al., 2016). To address these limitations, a publicly accessible experimental logBL database is needed to provide the quantitative data required for developing accurate BLB permeability prediction models. In the future, such datasets may become available through dedicated biomedical consortia or specialized ADME/Tox platforms that prioritize inner ear–focused research.

SNHL is a broad term used to describe various disorders that affect the functioning of the cochlea, the part of the inner ear that is dedicated to hearing. These disorders can result from genetic mutations, environmental factors, or age-related degeneration. Some of the most common types of peripheral auditory pathology include NIHL, ARHL, and ototoxicity. Indeed, many drugs that could theoretically cross the BLB have ototoxic side effects, which can exacerbate hearing loss rather than treat it. Various drugs often trigger oxidative stress, disrupt ion homeostasis, or induce direct cytotoxicity in cochlear hair cells, ultimately leading to irreversible hearing impairment (Lin et al., 2021).

Therefore, drug-induced ototoxicity can be evaluated via machine learning (ML) and deep learning (DL) models, similar to the consensus model, which is based on individual ML/DL, with total accuracies of 0.95 and 0.90, respectively (Huang et al., 2021).

An example of possible computational modeling is the oxidative stress at the BLB as a main factor of NIHL and ARHL, which is mediated via the Keap1-NRF2 pathway (Li et al., 2021; Oishi et al., 2020). In the context of this pathway, protein–protein molecular docking and molecular dynamics simulations can be used to understand the intermolecular affinity and conformational changes that occur in proteins. Keap1 is a substrate adaptor protein that binds to NRF2 and targets it for degradation. However, when a cell is exposed to oxidative stress, NRF2 is released from Keap1 and translocates to the nucleus, where it activates genes that protect against oxidative stress followed by hearing loss (Henderson et al., 2006).

The delivery of the gene encoding vesicular glutamate transporter 3 (VGLUT3) via AAV1 has been demonstrated to restore hearing function in mice lacking this protein (Akil et al., 2012). VGLUT3 is expressed in cochlear inner hair cells and holds promise as a potential vehicle for drug delivery across the BLB (Zhang et al., 2020).

For example, a drug could be designed to bind to VGLUT3, allowing it to be transported into the cochlea, where it could exert its therapeutic effects. By using VGLUT3 as a targeted delivery mechanism, drugs can be targeted to the inner ear, minimizing systemic exposure and reducing potential side effects. This approach could be useful for treating conditions such as NIHL, where direct delivery to the cochlea is challenging because of the restrictive nature of the BLB.

Hypothetically, the incorporation of glutamic acid as a moiety into the active pharmaceutical ingredient (API) could yield a prodrug form that enhances its permeation across the BLB via the glutamate–aspartate transporter. This transporter, which is highly expressed in the cochlea and plays a critical role in maintaining glutamate concentrations in the perilymph at nontoxic levels during acoustic overstimulation, could also facilitate drug permeation across the BLB. Importantly, the prodrug, which is initially pharmacologically inactive, undergoes metabolic conversion at the site of action, releasing the active drug. This targeted approach could not only improve the therapeutic efficacy of drugs intended for inner ear conditions but also minimize potential systemic side effects by limiting the exposure of nontarget tissues to the active drug.

As a result, computational methods such as molecular docking, molecular dynamics simulations, QSAR/QSPR analysis, transporter-enhanced BLB permeability prediction, high-throughput screening, and network pharmacology are essential but still need to be developed to provide novel therapies for SNHL. These cutting-edge methods can help identify potential drug candidates, predict their activity, optimize their properties, and analyze complex interactions between drugs, target molecules, and biological pathways.

### 6.2 Vagus nerve stimulation

**Figure 6:**
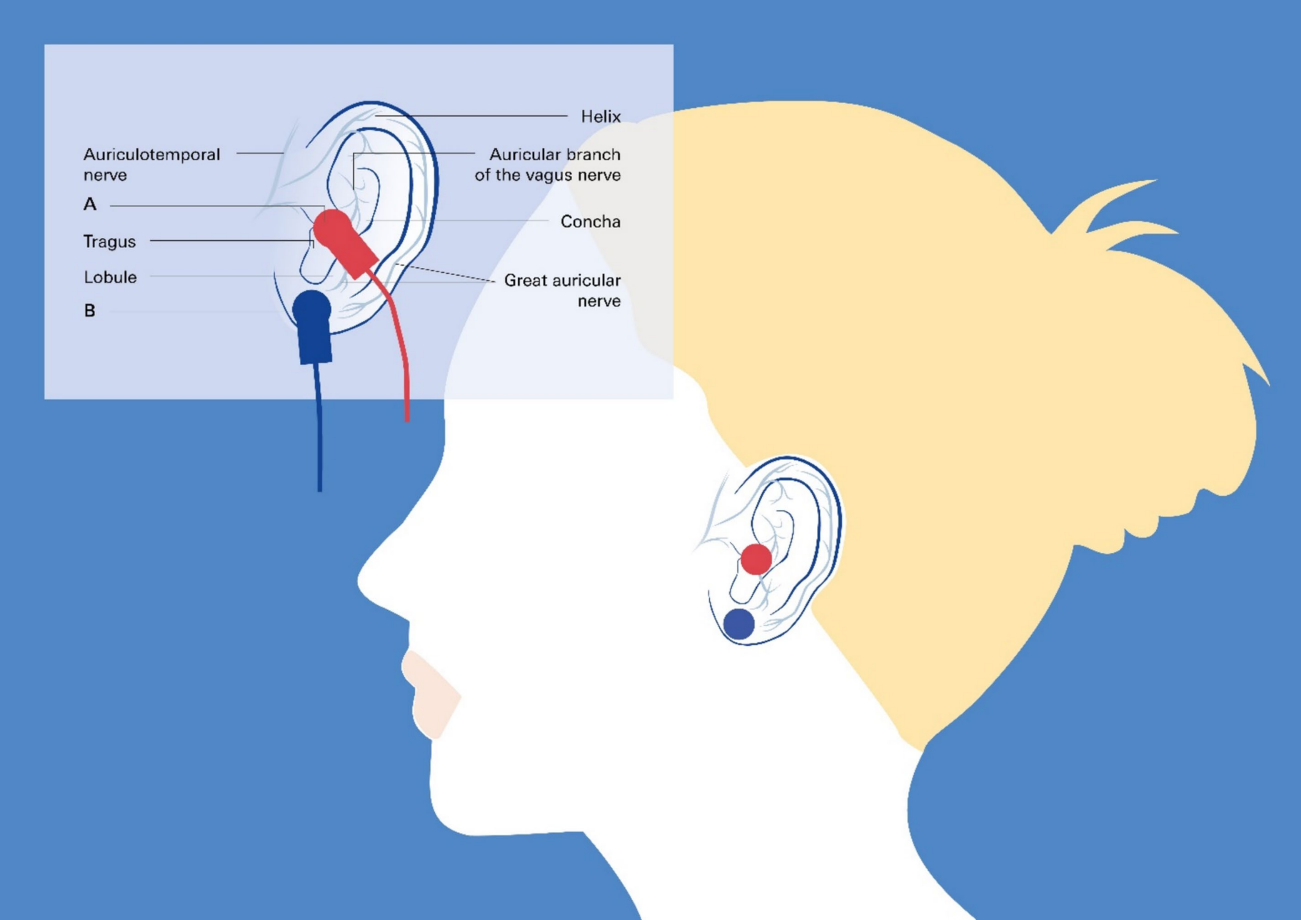
Transcutaneous vagus nerve stimulation targeting the auricular branch of the vagus nerve: taVNS. Noninvasive taVNS delivery systems rely on the cutaneous distribution of vagal fibers at the external ear (auricular branch of the vagus nerve) (Butt et al., 2020), as detailed in the insert. The red circles in the main image and the clamps in the insert represent the best anatomical sites for active left tragus stimulation by the taVNS device, and the blue circles and clamps represent the sham control stimulation sites.

The hypothesis that NIHL may lead to dampening of the vagus nerve implies that stimulation of the nerve may have a positive effect on NIHL. Therefore, an emerging therapeutic option could be to use vagus nerve stimulation (VNS) to treat NIHL. VNS is increasingly being used to treat conditions such as epilepsy, depression, and chronic inflammation, as well as an aid to rehabilitation and as a technique for cognitive improvement (Stefan et al., 2012; Ventureyra, 2000). Nonetheless, VNS requires implantation of a device via an invasive technique, which may not be acceptable for many patients (Zafeiropoulos et al., 2023). These limitations led to the development of transcutaneous VNS (tVNS) by stimulating the auricular branch of the vagus nerve at the tragus or the concha of the ear (Stavrakis et al., 2024; Hanna et al., 2021; Stavrakis et al., 2020; Tran et al., 2019). This technique is associated with minimal risk, thus opening up the possibility for novel applications even in healthy individuals (Figure 6).

taVNS in combination with tone therapy has been demonstrated to be an efficient and safe method that does not have significant side effects. Several preclinical and small cohort clinical studies support this assumption to combine taVNS with tones to improve auditory processing, e.g., in patients with tinnitus or possibly NIHL (Kochilas et al., 2020; Yakunina et al., 2018; Raj-Koziak et al., 2023; Suk et al., 2018).

With respect to acquired hearing loss following noise trauma, one could refer to reports in animals showing that VNS paired with specific tones improved tinnitus during follow-up and was sufficient to reverse the abnormal plasticity of the primary auditory cortex shown to be associated with tinnitus (Engineer et al., 2011). On the other hand, pilot clinical studies highlight the feasibility and safety of VNS paired with tones in patients with moderate to severe chronic tinnitus (Tyler et al., 2017). These studies combined with reported beneficial effects on hypertension (Annoni et al., 2015; Chapleau et al., 2016; Annoni et al., 2019; Nagai et al., 2023) and heart failure (Tran et al., 2019; Stavrakis et al., 2022), with minimal, if any, side effects, provide sufficient evidence that studies on this topic should be extended and moved forward.

Notably, noninvasive vagus nerve stimulation reduces BBB disruption in a rat model of ischemic stroke (Yang et al., 2018) such that a transferable effect to the strial BLB following NIHL can be assumed. VNS is known to reduce inflammation and oxidative stress, which are key factors in both BBB and BLB disruption. Given the functional similarities between the BBB and BLB, it is reasonable to hypothesize that VNS could protect the BLB in NIHL. However, direct experimental evidence is needed to confirm this effect, as the current assumption is based on indirect findings from stroke models. Further research is needed to validate the potential protective role of VNS in preserving BLB integrity in NIHL.

## 7 Conclusion

According to the 2020 World Report on hearing by the World Health Organization (Nieman and McMahon, 2020), disabling hearing loss affects more than 5% of the global population, with SNHL being a major contributor. SNHL can result from various factors, including vascular disorders, viral infections, ototoxic drugs, systemic inflammation, labyrinthine membrane degeneration, and NIHL. Prolonged exposure to loud sounds, typically above 85 dB, leads to permanent hearing loss and related health issues. In this work, advanced strategies and computational methods are explored for developing treatments for NIHL as well as its major comorbidity, hypertension, thereby offering potential solutions to this widespread problem. We recommend limiting exposure to noise to less than 85 dB in light of evidence showing that exposure above that threshold leads to autonomic changes and alterations in blood pressure (Wojciechowska et al., 2022; Thiesse et al., 2020; Gangwar et al., 2023). If exposure to levels above 85 dB occurs, concomitant taVNS treatment may dampen the adverse effects on autonomic tone and prevent hypertension. This notion needs to be studied in clinical trials.

A substantial amount of evidence is presented in this paper to demonstrate that computational methods and taVNS are suitable for developing future NIHL and hypertension treatments, thus providing possible solutions to this widespread issue.

## Glossary

ARHL: age-related hearing loss
BBB: blood–brain barrier
BLB: blood–labyrinth barrier
EP: endocochlear potential
HC: hair cell
LA: labyrinth artery
NIHL: noise–induced hearing loss
OoC: organ of Corti
PTS: permanent threshold shift
QSAR: quantitative structure–activity relationship
QSPR: quantitative structure–peritoneal relationship
RM: Reissner membrane
ScV: scala vestibule
SL: spiral ligament
SMA: spiral modular artery
SM: scala media
SNHL: sensorineural hearing loss
SSNHL: sudden sensorineural hearing loss
ST: scala tympani
SV: stria vascularis
taVNS: transcutaneous auricular vagus nerve stimulation
TTS: temporary threshold shift
tVNS: transcutaneous vagus nerve stimulation
