# Supplementary figures and images for: Interplay between noise-induced sensorineural hearing loss and hypertension: pathophysiological mechanisms and therapeutic prospects
Source: Front Cell Neurosci. 2025 Apr 7;19:1523149. doi: 10.3389/fncel.2025.1523149 (PMC12009814; doi:10.3389/fncel.2025.1523149)

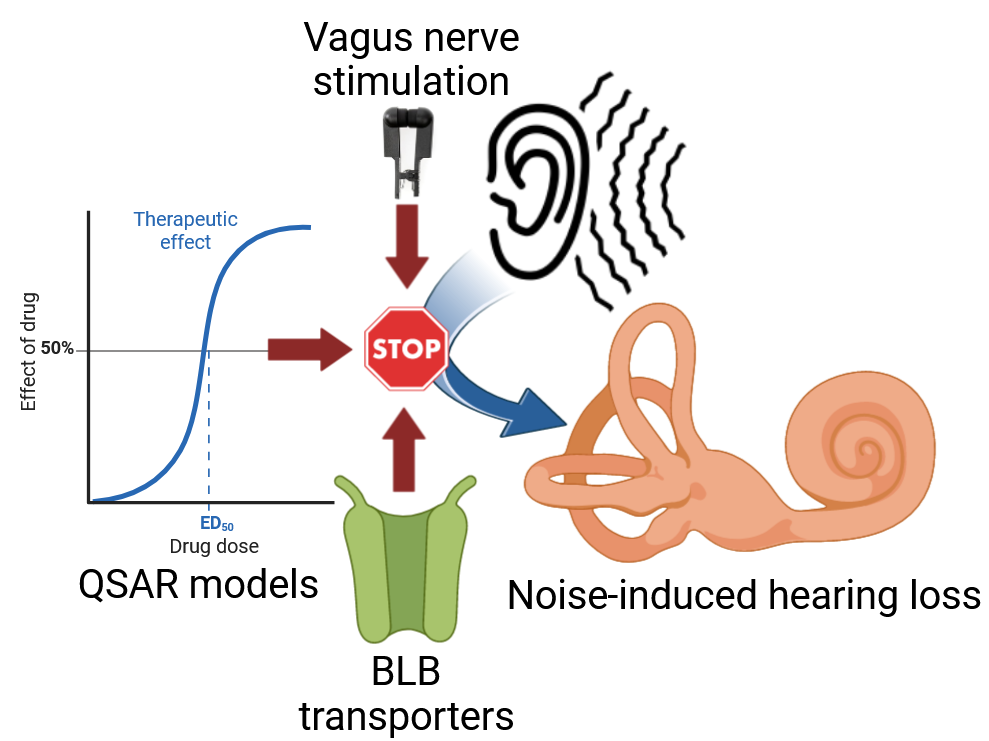

Supplement: Supplementary file 1 [file Image_1.PNG]
